# Clinical Features of Neuroblastoma With 11q Deletion: An Increase in Relapse Probabilities In Localized And 4S Stages

**DOI:** 10.1038/s41598-019-50327-5

**Published:** 2019-09-24

**Authors:** Antonio J Ribelles, Sandra Barberá, Yania Yáñez, Pablo Gargallo, Vanessa Segura, Bárbara Juan, Rosa Noguera, Marta Piqueras, Victoria Fornés-Ferrer, Jaime Font de Mora, Adela Cañete, Victoria Castel

**Affiliations:** 1 Pediatric Oncology and Hematology Unit, La Fe University Hospital, Valencia, Spain; 2 Medicine Faculty, Valencia, Spain; 3 Clinical and Translational Oncology Research Group, Investigation Institute La Fe, Valencia, Spain; 4 Pathology Department, Medicine Faculty. Hospital Clínico, Valencia, Spain; 5 Biostatistics Department, Investigation Institute La Fe, Valencia, Spain

**Keywords:** Prognostic markers, Molecular medicine, Paediatric cancer

## Abstract

Neuroblastoma (NB) is a heterogeneous tumor with an extremely diverse prognosis according to clinical and genetic factors, such as the presence of an 11q deletion (11q-del). A multicentric study using data from a national neuroblastic tumor database was conducted. This study compared the most important features of NB patients: presence of 11q-del, presence of *MYCN* amplification (MNA) and remaining cases. A total of 357 patients were followed throughout an 8-year period. 11q-del was found in sixty cases (17%). 11q-del tumors were diagnosed at an older age (median 3.29 years). Overall survival (OS) was lower in 11q-del patients (60% at 5 years), compared to all other cases (76% at 5 years) p = 0.014. Event free survival (EFS) was 35% after 5 years, which is a low number when compared with the remaining cases: 75% after 5 years (p < 0.001). Localized tumors with 11q-del have a higher risk of relapse (HR = 3.312) such as 4 s 11q-del patients (HR 7.581). 11q-del in NB is a dismal prognostic factor. Its presence predicts a bad outcome and increases relapse probability, specially in localized stages and 4 s stages. The presence of 11q aberration should be taken into consideration when stratifying neuroblastoma risk groups.

## Introduction

Neuroblastoma (NB) is a complex pediatric tumor and the most common extracranial solid malignancy in childhood^1^. Clinical manifestations may range from aggressive growth despite intensive treatment, to cases in which spontaneous regression is reported. In the last decades, it has been observed that particular tumoural genomic changes correlate with its behaviour and outcome. It is well known that an *MYCN* amplification (MNA) is associated with aggressive tumors and a dismal prognosis. Other genomic features besides MNA, such as 11q deletion (11q-del) and 17q gain, represent segmental aberrations that take place in a remarkable number of cases.

Segmental chromosomal aberrations (SCA) are considered a bad or negative prognostic factor. The frequency of 11q alterations may vary between 20–45%^1^, depending on the different series and study techniques used. These particular tumors have, in addition to segmental aberration in chromosome 11: a higher observed frequency of chromosomal breakage, a higher median age at diagnosis, and poor prognosis^2,3^ (survival rates estimated of 35% at 8 years). There is a poor amount of data comparing the outcome of 11q-deleted and MNA NB cohorts.

The international NB risk group (INRG) proposed a staging system in 2009 that stratified NB in risk groups according to clinical and genetic factors, such as 11q aberration in some particular stages^4^. Other factors taken into consideration were INRG stage (L1, L2, M, 4 s), age, tumor differentiation grade and histologic pattern, ploidy and *MYCN* status.

However, in some subgroups of the International Society of Pediatric Oncology European neuroblastoma research network (SIOPEN) (mainly intermediate and high risk cases), the presence of 11q aberrations is not taken into account when classifying the risk group, which may give rise to concern among the treating physicians, given the possibility that these patients are receiving insufficient treatment. To study in depth the characteristics and outcome of patients with 11q-deleted NB, a retrospective study has been conducted in a Spanish cohort comparing clinical features among 11q-del, MNA and other NB without any of these genetic abnormalities.

## Methods

Our aim is to study and describe the 11q-del NB cases diagnosed throughout an 8-year period. This retrospective multi-centric study consists of 399 NB patients from 29 Spanish cooperating hospitals. Data collected from patients included age at diagnosis, primary tumor location, pathology, stage, *MYCN* status, 11q-del status, relapse, time until relapse, time from relapse to death, cause of death, follow up and current state of the patient. Staging and risk stratification were established according to INSS and INRGSS classifications^5–7^. Tumor samples from all patients were referred to the Spanish reference center for pathology and molecular biology NB studies. Samples were centrally reviewed and classified according to the International NB Pathology Classification (INPC)^8–10^.

Patients were registered in the file of the Spanish Society of Pediatric Hematology and Oncology (SEHOP) NB group database, and included for treatment mainly in SIOPEN trials according to period and clinical characteristics (INES, LINES, LNESG-I, EUNS and HRNBL1). In stage M patients, response to induction treatment chemotherapy was evaluated according to SIOPEN guidelines (High Risk NB Study 1.7 of SIOP-Europe)^11^. Informed consent for study participation, samples and data management were obtained in all cases from the patients’ parents, and all the patients were treated following SIOPEN-approved protocols.

Chromosome 11q-del was defined as a missing (deleted) copy of genetic material on the long arm (q) of chromosome 11. *11q23* was the most frequent region found to be deleted. Biological studies included *MYCN* status and *1p* using FISH technique, and *11q* status was initially studied by multiplex ligation-dependent probe amplification technique (MLPA), and from 2012 onwards by single nucleotide polymorphism (SNP) array (Affymetrix and Illumina) according to the European Neuroblastoma Quality Assessment (ENQUA) guidelines (Ambros IM *et al*.)^12–14^.

As recommended by the ENQUA guidelines, amplification of *MYCN* was defined as a higher than a 4-fold increase of *MYCN* signals in relation to the number of reference chromosome 2 signals. The increase or the additional copies (up to the 4-fold) were defined as *MYCN* gain^15,16^.

The data used was summarized using mean, standard deviation (SD) and median (1^st^, 3^rd^ Q) in the case of continuous variables, whereas absolute and relative frequencies were used for categorical variables. To assess the independent association of 11q-del with survival, Cox proportional hazard regression models were adjusted including *MYCN* status, stage and age at diagnosis. For event free survival (EFS), time to event was defined as the time from diagnosis until the time of first occurrence of relapse, progression or death. For OS, time to event was defined as time until death or until last contact if the patient was alive. Kaplan-Meier curves were compared with log-rank test. Age difference between the presence and absence of 11q-del was compared using Wilcoxon Rank Sum test. 95% confidence Intervals of the effects were provided. Proportions were evaluated using Chi-square test. P-values below 0.05 were considered statistically significant. All analyses and graphs were performed using R software (version 3.5.0) with clickR (0.3.64) and survival (2.41–3) packages.

### Ethics approval and consent to participate

All study actions have been done under the appropriate ethics code and consent was obtained from all patients. The study was performed in accordance with the Declaration of Helsinki.

## Results

A total of 399 children with NB were registered during this 8-year study period (2006 to 2013). Tumors without initial 11q determination were excluded from analysis (42 patients). The remaining 357 NB were tested for 11q-del. 60/357 patients were found to have this alteration (17%). The presence of 11q-del and MNA in the same tumor was almost mutually excluding (only 3 cases showed both abnormalities).

The vast majority of the cases had abdominal location (79%), followed by the thoracic area (10%). A smaller proportion of tumors was found in the neck and in the pelvis, and there was one retro-orbital case. Concerning tumor stage, 177 patients (50%) had a localized disease (stage 1, 2 or 3), 145 patients were diagnosed as stage 4 (40%) and 35 patients had stage 4 s NB (10%). Median age at diagnosis was 1,37 years.

According to the INPC system^4^ (International NB Pathology Classification), 202 samples were classified as poorly differentiated NB (57%) leading this to be the most frequent diagnosis, followed by undifferentiated NB (14%). MNA was found in 63 patients (18% of the cases), whilst 38 tumors had *MYCN* gain (10%).

From the whole cohort, 105 patients died (30%) and 252 are alive with a median follow up of 5,7 years. The most important cause of death was tumor progression in 90% of the cases. Regarding the survivors, 222 patients remain free of disease (62%) and 30 patients are alive on treatment after relapse (8%). Table 1 summarizes the clinical and biological characteristics of the patients.

**Table 1:** General description of the studied population.

Variable	n = 357
Age at diagnosis (years)	mean: 2.51
	median: 1.37
Location	
Abdominal	281 (79%)
Neck	8 (2%)
Neck-thoracic	6 (1.5%)
Pelvic	6 (1.5%)
Retro-orbital	1 (0.3%)
Thoracic	35 (10%)
Thoracic-abdominal	20 (6%)
11q-del	
No	297 (83%)
Yes	60 (17%)
MNA	
Amplified	63 (18%)
Gained	38 (10%)
Non amplified	256 (72%)
Stage	
1	85 (24%)
2	25 (7%)
3	67 (19%)
4	145 (40%)
4 s	35 (10%)
Pathology	
GanglioNB	41 (11%)
Non specified GanglioNB	6 (2%)
Differentiating NB	17 (5%)
Undifferentiated NB	52 (14%)
Poorly differentiated NB	202 (57%)
Anaplastic NB	2 (1%)
Non specified NB	37 (10%)
Current state	
Dead	105 (30%)
Alive with disease	30 (8%)
Alive without disease	222 (62%)
Cause of death	
Disease progression	94 (90%)
Other causes	11 (10%)

**Figure 1:**
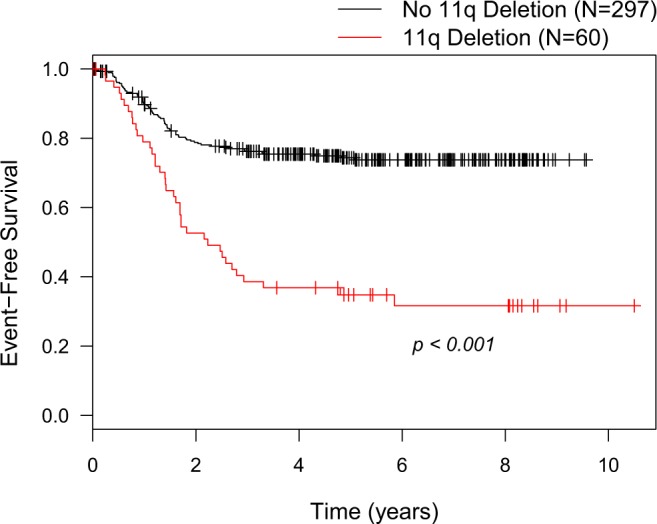
Event-free survival of 11q-del compared to no-11q-del neuroblastoma.

Initially, 11q status was considered as a unique factor and 11q-del population (n = 60) was compared to the no-11q-del cases (n = 297) including all age groups and stages. Overall survival (OS) was lower in 11q-del patients (60% at 5 years and 52% at 8 years) than in the cases lacking 11q-del (76% at 5 years and 72% at 8 years) p = 0.014. Differences were noticeable when considering EFS. In 11q-del patients, EFS was 35% at 5 years and 32% at 8 years compared to the data in no-11q-del cases: 75% at 5 years and 73% at 8 years (p < 0.001) with a median follow up of 5.7 years. (Fig. 1).

**Figure 2:**
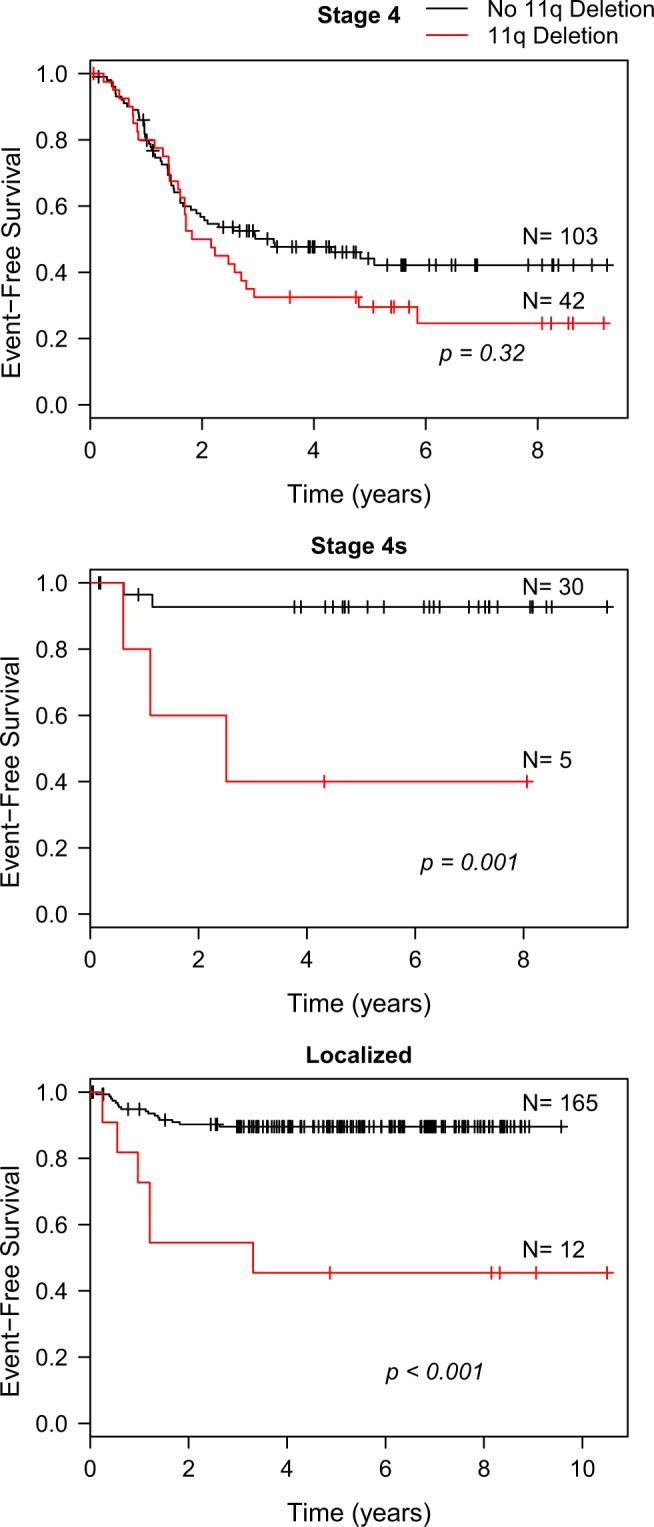
Event-free survival analysis by stages (stage 4, 4 s and localized) comparing 11q-del vs no-11q del NB cases.

Separating the data by stages (4, 4 s or localized), it was observed that localized and 4 s stages 11q-del patients had a poor EFS (approximately 45% and 40% respectively) (Fig. 2). EFS was similar in 11q-del localized or 4 s stages to no-11q-del stage 4 cases. This fact is very representative and further confirms that 11q-del status plays an important role in NB relapse. Statistically significant differences were observed when comparing 11q-del and non-11q-del EFS in localized NB and stage 4 s (p = 0.001). Differences in stage 4 EFS were observed according to 11q-del status although this result was not statistically significant (p = 0.32).

**Figure 3:**
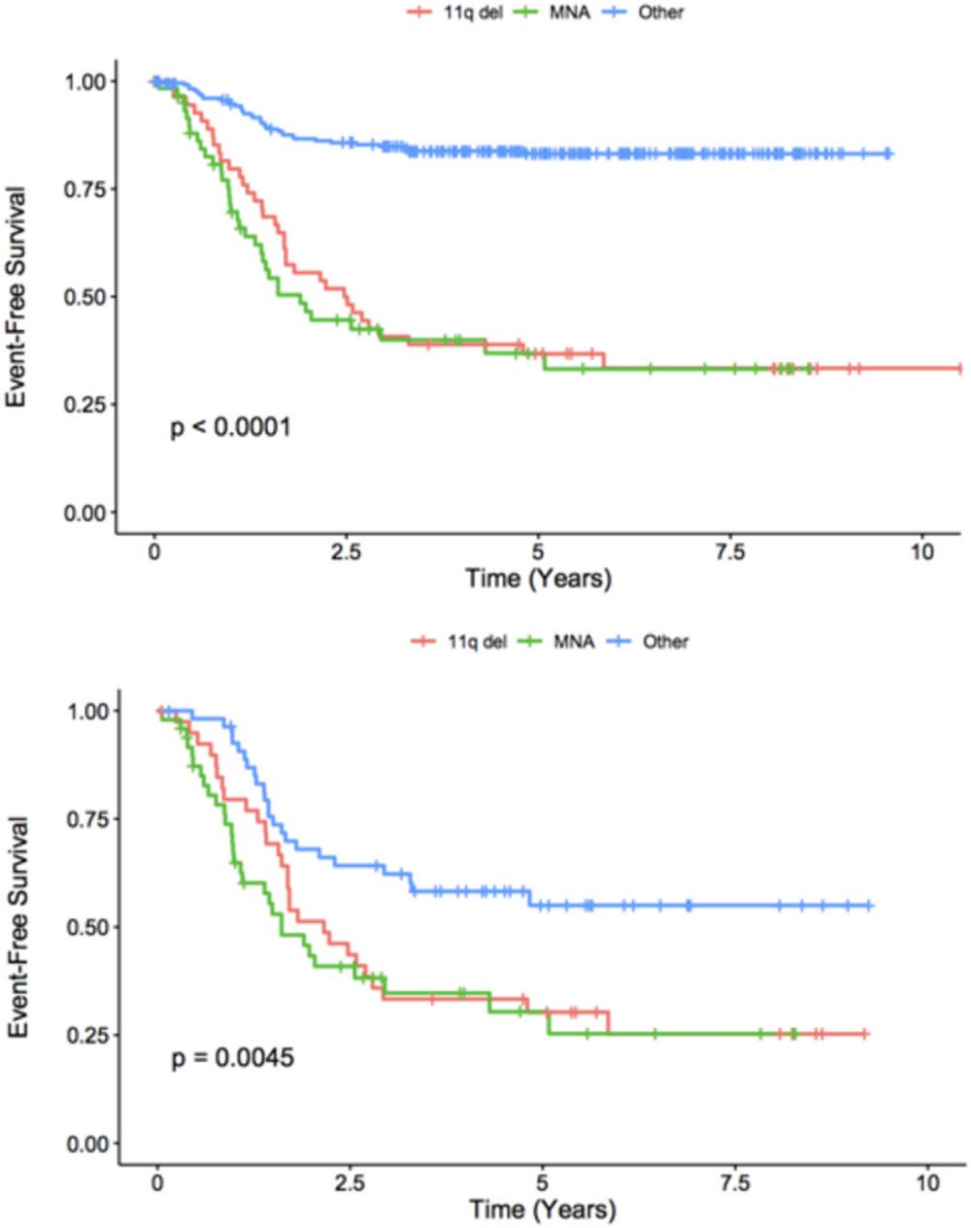
Kaplan-Meier curve comparing EFS between 11q-del, MNA and the remaining cases of NB. (Above: Entire cohort. Below: Stage 4 NB).

Given the importance of MNA as an established prognostic factor associated to adverse outcome in neuroblastoma, a comparison between EFS in MNA, 11q-del and the remaining NB cases without these alterations was made using Kaplan-Meier curves. Statistically significant results were found and no differences between MNA and 11q-del outcomes were observed (Fig. 3; above: Entire cohort; below: Stage 4 NB).

In the study, three cohorts were compared: 57 patients with 11q-del, 60 patients with MNA, and other 237 cases without both alterations (other population). The only 3 cases containing both abnormalities were excluded. The general characteristics of these subgroups are summarized in Table 2 and are the following.

**Table 2:** Description of the three neuroblastoma subgroups: 11q-del, MNA and the remaining cases.

	11q del n = 57	MNA n = 60	Other = 237
Median age at diagnosis (years)	3.29	2.02	0.92
Stage			
1	3 (5%)	1 (2%)	80 (34%)
2	0 (0%)	0 (0%)	24 (10%)
3	7 (13%)	9 (15%)	51 (22%)
4	42 (73%)	48 (80%)	55 (23%)
4 s	5 (9%)	2 (3%)	27 (11%)
Number of relapses			
0	21 (37%)	26 (43%)	198 (84%)
1	26 (46%)	33 (55%)	31 (13%)
2 or more	10 (17%)	1 (2%)	8 (3%)
Death			
No	32 (56%)	18 (30%)	203 (85%)
Yes	25 (44%)	42 (70%)	34 (15%)

### Age

Median age at diagnosis was found to be higher in the 11q cases (3.29 years at diagnosis) when compared with MNA cohort (2.02 years) and the remaining cases (0.92 years at diagnosis).

### Stage

When comparing tumor stage, 42 cases (73%) of 11q-del patients were classified as stage 4 at diagnosis. This figure is similar to the number of MNA stage 4 cases (80%) while only 55 cases out of 237 (23%) of the other NB cases were classified as stage 4.

### *MYCN* gain

It was observed that out of sixty 11q-deleted NB, 20 had also *MYCN* gain (33.3%) whereas only 18 of the other 237 cases (without 11q-del) had *MYCN* gain (7.6%). These results are statistically significant (p < 0.001) and show that despite 11q-del and MNA being almost mutually excluding, 11q-del is associated with *MYCN* gain. Most of the patients with both these aberrations (11q-del and *MYCN* gain) presented high-risk features, 16/20 were diagnosed as stage 4 and 15/20 were older than 18 months.

### Relapse

A small number of tumor relapses were reported in the cohort without MNA or 11q alterations (relapses in 16% of the cases). On the other hand, more MNA or 11q-del NB recurred. 55% of the MNA tumors had one relapse and one case had two recurrences. Among the 11q-del cases, 36/57 patients relapsed (63%). 26 patients relapsed only once (46%) and ten cases with sequential relapses were detected (6 patients had two relapses, 2 patients had three relapses and 2 patients recurred four times) (Table 2).

In the relapse model (Cox regression), the population with higher relapse risk was stage 4 s with 11q-del, with 7.581 HR (hazard ratio). *MYCN* homogeneous amplification was also associated with a higher relapse risk (HR 2.718). Localized tumors (stage 1, 2, 3) with 11q-del also have a higher risk (HR 3.312). Notably, 4 s population and localized NB without 11q-del had the lowest HR (0.245 in both cases) showing how aberrations in 11q dramatically increase relapse rate in low risk NB. These results are statistically significant. Table 3 summarizes relapse data in these subgroups of patients.

**Table 3:** Relapse risk in neuroblastoma subgroups (Cox regression relapse model).

Relapse	HR	Lower 95%	Upper 95%	P-value
Stage 4 s	0.245	0.056	1.065	0.061
Localized	0.245	0.133	0.449	<0.001
11q-del	1.847	1.087	3.138	0.023
*MYCN* gain	1	0.573	1.745	0.999
*MYCN* amplification	2.718	1.672	4.421	<0.001
Age < 18 months	0.405	0.252	0.653	<0.001
Stage 4 s with 11q del	7.581	1.175	48.924	0.033
Localized with 11q del	3.312	1.128	9.727	0.029

The clinical course of 11q-del NB patients was usually an insidious process with multiple relapses and longer courses with a poor final outcome, based on time until relapse, time from relapse to death and time from diagnosis to death. Patients with 11q-del relapsed later than the other cases. Median time from diagnosis to relapse was 1.42 years, compared with 1.08 in the MNA cohort and 1.28 in the other NB relapsed patients. Relapsed 11q-del patients also had a longer median time from relapse to death (1.53 years) than MNA cases (0.49 years) and than the other NB cases (0.98 years). Finally, median time from diagnosis to death was longer in 11q-del patients (2.88 years) compared to MNA NBs (1.37 years) and the remaining patients (2.09 years).

### Survival/event free survival

Statistical differences were observed in OS when comparing patients in the three groups. Mortality is higher in the MNA NB with 70% of deaths (42/60 compared with 44% in the 11q-del tumors (25/57) and 15% in the remaining patients (34/237). MNA patients had a HR of 3.673 (p < 0.001). Therefore, by using Cox regression we conclude that MNA amplification was associated with a lower survival, a fact that has been widely identified (Table 4).

**Table 4:** Death risk in neuroblastoma subgroups (Cox regression survival model).

Death risk	HR	Lower 95%	Upper 95%	P-value
Stage 4 s	0.391	0.112	1.363	0.14
Localized	0.185	0.093	0.37	<0.001
11q del	1.145	0.648	2.02	0.641
*MYCN* gain	1.251	0.689	2.273	0.462
*MYCN* amplification	3.673	2.243	6.015	<0.001
Age < 18 months	0.352	0.209	0.594	<0.001
Stage 4 s with 11q del	4.398	0.681	28.409	0.12
Localized with 11q del	3.119	0.866	11.227	0.082

The most important differences in 11q-del NB were observed in final outcome in localized and stage 4 s tumors. Localized stages with 11q-del showed higher risk of death (HR 3.119, p = 0.082). Moreover, stage 4 s patients with 11q-del presented a HR of 4.398, p = 0.12). These results were not statistically significant, probably due to the low number of 11q-del localized and stage 4 s cases. Even so, given the notoriousness of these values, they should be taken into account.

## Discussion

During the last years many international efforts have been done trying to investigate why some particular genetic changes lead to more aggressive cases of NB. MNA is still the most relevant biological prognostic factor, specially in infants^17^. However, in the last years, SCA including *11q* alterations are also taken into account and affect risk stratification in some subgroups of NB. This is one of the largest cohorts (n = 357) that shows and further confirms worst outcomes in NB containing 11q-del. Patients have been studied homogeneously without previous selection and have a median follow up of 5.7 years. As it has been described, 11q-del NB is related to older age at diagnosis (p < 0.001) and is also associated with more advanced stages of NB (p < 0.001). Co-presence of 11q-del and MNA is extremely rare. Using Cox regression, we conclude that 11q-del as well as MNA is associated with a higher risk of relapse. The comparison between EFS in MNA, 11q-del and the remaining cases with Kaplan-Meier curves (Fig. 3) further confirms the clinical value of 11q-del in NB, being equivalent to that of MNA.

In the cohort studied previously by Schleiermacher *et al*., it was observed that in 147 NB without MNA, a SCA profile was the strongest independent prognosis factor. In this cohort, 76% of the cases with SCA showed 11q-del^4^. Caren *et al*., reported that median age at diagnosis was significantly higher in the 11q-del group compared to numeric chromosomal aberrations (NCA), MNA and 17q gain groups (42 months vs 3, 21 and 21 months, respectively). Prognosis was found to be poor in MNA and 11q-del groups (8 years OS 35%), but the median survival time after diagnosis was longer in 11q-del NB (40 vs 16 months)^1,3^. These observations are very similar to our findings as 11q-del NB patients in our study have shown higher median time to relapse, higher median time from relapse to death and higher median time from diagnosis to death.

Concerning age, similar conclusions have been reported by previous groups highlighting that 11q alteration is detected mostly in older patients. Cetinkaya C *et al*. reported a cohort of NB where median age at diagnosis was extremely different according to MNA or 11q-del. The results were 58.5 months in 11q-del NB vs 18 months in the MNA group^1,18^. Analysis of the INRG database has also shown that in a cohort of younger patients (<18 months) with stage 3 NB, the only independently associated factor with poor survival and EFS has been 11q-del status. These facts are completely concordant with our results^1,19^. Adolescents with neuroblastoma represent less than 5% of the cases and in most series they are characterized by a high prevalence of SCA and a low incidence of MNA. The prevalence of 11q-del in this group is between 30 to 60%^1^. ALK and ATRX mutations are more frequent in older patients too and ATRX mutated NB showed a higher number of SCA including 11q-del^20,21^ with a very poor outcome.

We also report that 11q-del is associated to *MYCN* gain. The proportion of *MYCN* gain in the 11q-del cohort is much higher than in other cases (33.3% versus 6.1%). Our data also confirms the association between 11q aberration and high-risk disease, specifically in the absence of MNA. *MYCN*-gain most likely occurs due to a larger gain of copies of the 2p chromosomal arm, rather than a focal gain, meaning that additional genes at 2p including ALK could contribute to NB pathogenesis and high risk disease. Furthermore, recent findings have provided a potential link between this inverse association between 11q aberration and MNA. More specifically, evidence has suggested that dysregulation of the microRNA *let-7* plays a central role in the pathogenesis of neuroblastoma and that either MNA or 11q loss are able to disrupt *let-7*, but the biological significance of this relationship is still waiting to be confirmed^22^.

In the subset of patients with “good prognosis” (localized and 4 s stages), 11q-del frequency is rather low. However, when these cases have 11q-del, prognosis has shown to be worse^23^. Hence, some authors support that patients in low and intermediate risk groups with SCA such as 11q-del could benefit of intensified treatments^24^. In the COG protocols, 11q-del is added to other factors (*MYCN* status and ploidy) in patients with localized tumors younger than 18 months. In SIOPEN studies, chromosomal segmentation aberrations are considered including 11q-del but these are not considered in all tumor stages. Currently, it is well recognized that 11q-del NB constitutes a distinct subgroup of aggressive malignancies, but with different features compared to MNA and therefore, the results show that some action points regarding treatment need to be further assessed in this field. A high frequency of chromosomal breakage, suggestive of a chromosomal instability, is one of the main features of 11q-del NB that has been previously reported. This shows that certain genes in 11q could be involved in the chromosomal instability phenotype^3^, by haplo-insufficiency or inactivation of the second allele by mutation or epigenetic modification^1^. Recurrent patterns of SCA or NCA suggest that NB is a cancer driven by copy number rather than by particular mutations. The fact that 11q is never lost on both chromosomes suggests that important genes are present on the remaining 11q copy, but that the second hit needed would be caused by another localized mutation or methylation event^1^.

So far, the mechanism by which hemizygous deletion in 11q leads to high risk features is unknown, and therapies targeting this alteration have not been totally developed. Some of the genes located in this chromosomal area have been studied and seem to have important relation with the adverse prognosis that the ablation produces. Particularly, TSLC1 (CADM1; cell adhesion molecule 1) located in 11q23.3 has an important role as tumor suppressor gene in NB and has also been related in oncogenesis^25^. Recently, while looking for new possibilities and alternative therapies in patients with relapsed 11q-del tumors, ATM hemizygosis (11q22.3) in the presence of functional TP53 (17p13.1) has shown *in vitro* and *in vivo* response to PARP inhibitors^26^.

The gene ATM is within this chromosomic locus and has the role of repairing DNA damage by homologous recombination. Efficient repair of damaged DNA strands helps maintain the stability of the cell’s genetic information^27^. H2AFX (H2A Histone Family Member X) also located in 11q23.3 is a member of the nucleosome structure and thereby plays an important role in transcription regulation, chromosomal stability, DNA repair and replication. In fact, ATM protein phosphorylates H2AFX during response to double-strand breaks (DSB). Therefore the loss of H2AFX also suggests a potential utility of PARP inhibitors and could be related in the described responses^26,28^.

Poly ADP-ribose polymerase (PARP) is a protein that signals DNA damage and facilitates DNA repair. PARP catalyzes the addition of ADP-ribose to DNA, histones, topoisomerases and helicases and has a critical function in cellular replication, transcription, differentiation, gene regulation, protein degradation and spindle maintenance. Inhibition of PARP results in persistent single strand DNA breaks leading to stalled replication forks and double strand DNA breaks. PARP inhibition produces DNA damage that leads to cell cycle arrest and apoptosis. PARP inhibitors are being evaluated in cancers with defective DNA repair mechanisms alone or in combination with cytotoxic therapy or radiation^29,30^. The addition of PARPi to second line chemotherapy in 11q-del neuroblastoma patients could be an attractive combination for these patients that is currently under exploration.

In the basis of these facts and other similar hypotheses of previous revisions, we think that within the international groups, new frontline strategies are required to be developed in order to improve the outcome of neuroblastoma patients with 11q-del.

## Data Availability

Data and materials are available in the national neuroblastic tumor data base in University Hospital La Fe.
