# Publisher Correction: Clinical Features of Neuroblastoma With 11q Deletion: An Increase in Relapse Probabilities In Localized And 4S Stages

**DOI:** 10.1038/s41598-020-58428-2

**Published:** 2020-01-31

**Authors:** Antonio Juan Ribelles, Sandra Barberá, Yania Yáñez, Pablo Gargallo, Vanessa Segura, Bárbara Juan, Rosa Noguera, Marta Piqueras, Victoria Fornés-Ferrer, Jaime Font de Mora, Adela Cañete, Victoria Castel

**Affiliations:** 1 Pediatric Oncology and Hematology Unit, La Fe University Hospital, Valencia, Spain; 2 Medicine Faculty, Valencia, Spain; 3 Clinical and Translational Oncology Research Group, Investigation Institute La Fe, Valencia, Spain; 4 Pathology Department, Medicine Faculty. Hospital Clínico, Valencia, Spain; 5 Biostatistics Department, Investigation Institute La Fe, Valencia, Spain

Correction to: *Scientific Reports* 10.1038/s41598-019-50327-5, published online 24 September 2019

The original version of this Article contained a typographical error in the spelling of the author Antonio Juan Ribelles, which was incorrectly given as Antonio J Ribelles.

As a result, the Author Contributions statement,

“Antonio J Ribelles and Adela Cañete: Main authors. Wrote manuscript text. Sandra Barberá and Bárbara Juan: Data collection and english translation. Yania Yáñez, Pablo Gargallo and Victoria Castel: Wrote parts of the manuscript. Vanessa Segura, Rosa Noguera, Marta Piqueras and Jaime Font de Mora: Molecular testing. Sample management. Victoria Fornés-Ferrer: Biostatistical analysis.”

now reads:

“Antonio Juan Ribelles and Adela Cañete: Main authors. Wrote manuscript text. Sandra Barberá and Bárbara Juan: Data collection and english translation. Yania Yáñez, Pablo Gargallo and Victoria Castel: Wrote parts of the manuscript. Vanessa Segura, Rosa Noguera, Marta Piqueras and Jaime Font de Mora: Molecular testing. Sample management. Victoria Fornés-Ferrer: Biostatistical analysis.”

These errors have now been corrected in the PDF and HTML versions of the Article.

